# A single-center, double-blind, randomized, placebo-controlled, two-arm study to evaluate the safety and efficacy of once-weekly sirolimus (rapamycin) on muscle strength and endurance in older adults following a 13-week exercise program

**DOI:** 10.1186/s13063-024-08490-2

**Published:** 2024-10-01

**Authors:** Brad Stanfield, Matt Kaeberlein, Brian Leroux, Julie Jones, Ruth Lucas, Bruce Arroll

**Affiliations:** 1 Royal New Zealand College of General Practitioners, Wellington Central, Wellington, 6011 New Zealand; 2 University of Auckland, Auckland, New Zealand; 3 Optispan, Inc., Seattle, WA USA; 4 Department of Oral Health Sciences, University of Washington, Seattle, WA USA; 5 Department of Biostatistics, University of Washington, Seattle, WA USA; 6 BioValeo, Wellington, New Zealand; 7 General Practice and Primary Health Care, University of Auckland, Auckland, New Zealand

**Keywords:** Aging, Muscle strength, Muscle endurance, Sirolimus, Rapamycin, mTOR, Sarcopenia, Older adults, Randomized controlled trial, Exercise

## Abstract

**Background:**

Aging leads to a decline in muscle mass and strength, contributing to frailty and decreased quality of life. Sirolimus (rapamycin) , an mTOR inhibitor, has shown potential in preclinical studies to extend lifespan and improve health span. This study evaluates the safety and efficacy of once-weekly sirolimus (rapamycin) administration on muscle strength and endurance in older adults engaged in a 13-week exercise program.

**Methods:**

This randomized, double-blind, placebo-controlled trial will enroll 40 participants aged 65–85. Participants will be randomly assigned to receive either sirolimus (rapamycin) 6 mg/week or placebo for 13 weeks, in conjunction with an at-home exercise program. The primary outcome measure is the change in muscle strength and endurance, assessed by the 30-Second Chair-Stand Test. Secondary outcome measures include adverse events, changes in muscle strength and endurance as measured by the 6-min walk test, handgrip strength, and participant-reported outcomes using the SF-36 survey. Assessments will be conducted at baseline, mid-intervention (week 6), and post-intervention (week 13). Blood samples will be collected for hematology and biochemistry analyses, including full blood count, urea and electrolytes, liver function tests, HbA1c, lipids, serum IGF-1, and hs-CRP. DNA methylation will be analyzed using TruDiagnostic™ to explore changes in biological age.

**Discussion:**

This study aims to provide insights into the potential benefits of intermittent sirolimus (rapamycin) administration on muscle performance in older adults. By alternating periods of mTOR inhibition through rapamycin and activation via exercise, this study will explore a novel approach to enhancing muscle strength and endurance in the aging population. The results could have significant implications for developing interventions to improve physical function and overall health outcomes in older adults. Safety and tolerability will also be closely monitored to ensure the feasibility of this regimen for wider application.

**Trial registration:**

Australia New Zealand Clinical Trials Registry, ACTRN12624000790549. Registered on 26 June 2024 https://www.anzctr.org.au/Trial/Registration/TrialReview.aspx?ACTRN=12624000790549.

## Administrative information

Note: the numbers in curly brackets in this protocol refer to SPIRIT checklist item numbers. The order of the items has been modified to group similar items (see http://www.equator-network.org/reporting-guidelines/spirit-2013-statement-defining-standard-protocol-items-for-clinical-trials/).

**Table:** 

Title {1}	A single-center, double-blind, randomized, placebo-controlled, 2-arm study to evaluate the safety and efficacy of once-weekly Sirolimus (Rapamycin) on muscle strength and endurance in older adults following a 13-week exercise program. Trial acronym: RAPA-EX-01
Trial registration {2a and 2b}.	Australia New Zealand Clinical Trial Registry: ACTRN12624000790549
Protocol version {3}	Protocol version: Original (V1.0) Protocol date: 17^th^ June 2024
Funding {4}	Dr. Brad Stanfield Ltd, owned by the lead author Brad Stanfield, has funded this study along with Vitasang Ltd. Brad Stanfield has no affiliation with Pfizer (the manufacturer of Sirolimus) and no financial incentive in the outcome of this study. He is a General Practitioner in Auckland, New Zealand, with an active interest in preventative care. To ensure separation between the sponsor and the conduct of the trial, Aotearoa Clinical Trials Trust will be conducting the study, and Dr. Brad Stanfield Ltd will have no influence on data collection. Vitasang Ltd had no role in the design of this study and will not have any role during its execution, analyses, interpretation of the data, or decision to submit results. Sirolimus (Rapamycin) will be purchased from Pfizer, who have no input into the trial design. CompoundLabs will prepare the placebo and Sirolimus (Rapamycin) blister packs and label them with study identifiers. Rutherford Fitness is providing the exercycle bikes. Additionally, Aotearoa Clinical Trials Trust will be conducting the trial to ensure separation between the sponsor and the conduct of the trial. There are no conflicts of interest to declare.
Author details {5a}	Dr. Brad Stanfield conceived of the study and initiated the design. He is a Fellow of the Royal New Zealand College of General Practitioners, and affiliated with the University of Auckland. Matt Kaeberlein helped to refine the study design. He is the Chief Executive Officer of Optispan, Inc and an Affiliate Professor of Oral Health Sciences at the University of Washington. Brian Leroux is a Biostatistician and Professor at the University of Washington. He contributed to the study design and will conduct the statistical analyses. Julie Jones contributed to study quality by design, and study oversight and management. She is the CEO of BioValeo, the CRO assigned study delivery responsibility by the Sponsor. Ruth Lucas contributed to study quality by design, and study monitoring and management. She is the COO of BioValeo, the CRO assigned study delivery responsibility by the Sponsor. Bruce Arroll has helped design the study design and is a Professor of General Practice and Primary Health Care at the University of Auckland All authors contributed to refinement of the study protocol and approved the final manuscript
Name and contact information for the trial sponsor {5b}	Primary Trial Sponsor: Dr. Brad Stanfield Ltd Contact name: Brad Stanfield Address: 111 Newton Road, Eden Terrace, Auckland, 1010 , New Zealand Telephone: +64210426045 Email: brad@drstanfield.com Secondary Trial Sponsor: Vitasang Ltd Contact name: Scott King Address: 2340 Chartres Street, New Orleans, LA 70117, USA Email: info@vitasang.com
Role of sponsor {5c}	Dr. Brad Stanfield Ltd, owned by the lead author Brad Stanfield, has funded this study along with Vitasang Ltd. Brad Stanfield has no affiliation with Pfizer (the manufacturer of Sirolimus) and no financial incentive in the outcome of this study. He is a General Practitioner in Auckland, New Zealand, with an active interest in preventative care. To ensure separation between the sponsor and the conduct of the trial, Aotearoa Clinical Trials Trust will be conducting the study, and Dr. Brad Stanfield Ltd will have no influence on data collection. Vitasang Ltd had no role in the design of this study and will not have any role during its execution, analyses, interpretation of the data, or decision to submit results. BioValeo Ltd will act as the Contract Research Organisation (CRO) providing independent study design, management and monitoring services. Julie Jones and Ruth Lucas, from BioValeo, have no affiliation with Pfizer and no financial incentive in the outcome of this study.

## Introduction

### Background and rationale {6a}

#### Brief mechanistic hypothesis

Periods of time where the mechanistic target of rapamycin (mTOR) pathway is activated via exercise, combined with alternate periods of time where mTOR is inhibited using rapamycin (sirolimus), will result in greater muscle performance in older adults compared with just exercise alone. 

#### Background

Human skeletal muscle undergoes significant changes with aging, characterized by a decline in muscle mass and strength at an approximate rate of 1% per year starting around the age of 40 [1]. This decline in strength is inversely associated with all-cause and cardiovascular mortality [1]. Muscle wasting contributes to weakness, disability, increased hospitalization, immobility, and loss of independence. Interventions for sarcopenia, including exercise and nutrition, positively impact protein anabolism and enhance physical function, quality of life, and an anti-inflammatory state in older adults [1].

#### Sirolimus (rapamycin)

Sirolimus (rapamycin), also known as Rapamune, is a natural macrocyclic lactone produced by the bacterium *Streptomyces hygroscopicus*. It is FDA-approved for conditions like immunosuppression to prevent organ transplant rejection and inhibition of vascular smooth muscle cell proliferation in drug-eluting coronary stents. It is also used in treating various cancers and lymphangioleiomyomatosis due to its antiproliferative effects [2].

#### Mechanistic target of rapamycin (mTOR)

mTOR is a protein kinase belonging to the phosphoinositide 3-kinase (PI3K)-related kinase family, forming two types of multiprotein complexes: mTORC1 and mTORC2 [3, 4]. Both complexes play vital roles in cellular regulation, and their dysregulation disrupts cell homeostasis, potentially leading to aging-related pathologies. Sirolimus (rapamycin) is a specific allosteric inhibitor of mTORC1, which plays critical roles in regulating mRNA translation, promoting lipid and nucleotide synthesis, and repressing catabolism and autophagy [5]. mTORC1 is highly sensitive to sirolimus (rapamycin), while mTORC2 is only inhibited after prolonged exposure [5].

#### Preclinical evidence

There is abundant preclinical evidence that mTORC1 activity is a major driver of the biological aging process. In multiple organisms, genetic or pharmacological inhibition of the mTORC1 pathway has been shown to extend life and broadly delay molecular and functional declines associated with aging. These include genetic studies demonstrating that depleting mTOR or Raptor (a subunit of mTORC1) has life-extending effects in yeast [6], nematodes [7, 8], flies [9], and mammals [10], and similar outcomes when each of these organisms are treated with sirolimus.

- Mouse: sirolimus (rapamycin) treatment increases median and maximum lifespan when started at middle age (270 of 600 days of age) [11]. Treatment for only 3 months at middle age was sufficient to increase life expectancy by up to 60% and improve measures of health span in middle-aged mice [12]. Furthermore, sirolimus (rapamycin) treatment in mice was shown to increase alveolar bone levels [13], improve cardiovascular function [14–16], decrease body weight [17], and remodel the microbiome [12].
- Rat: sirolimus (rapamycin) treatment reduces food intake and body weight [16], increases grip strength, and attenuates the decline in maximum running distance [18]. Daily dosing in food did cause some diabetes-like symptoms [18], but repeated low doses do not seem to influence glucose homeostasis with long-term use [16].
- Dog: a study by Urfer et al. showed that low-dose (0.05 mg/kg and 0.10mg/kg) sirolimus (rapamycin) treatment three times a week over a period of 10 weeks in middle-aged dogs was well tolerated, with no clear adverse effects except for a decrease in the volume of red blood cells. The dogs had improvements in their left ventricular cardiac function and had increased activity [19].

#### Clinical evidence

- FDA-approved uses: sirolimus is approved for immunosuppression in organ transplant patients and cancer treatment, with higher doses associated with significant side effects including stomatitis, diarrhea, nausea, anemia, cytopenia, hyperglycemia, and hyperlipidemia, as well as the (intended) immunosuppressive effect.
- Low-dose studies: lower doses of sirolimus have shown safety in older populations. Studies have demonstrated well-tolerated doses with few mild side effects and improved immune function in elderly subjects [20–25].
- Exercise interaction: a study in young males found that sirolimus taken before resistance exercise impaired mTORC1 signaling and muscle protein synthesis, indicating the importance of timing sirolimus administration on non-exercise days [26].

#### Selection of orally bioavailable rapamycin analogue

Although structurally similar, sirolimus and everolimus do not have identical effects on the mTOR pathway. Sirolimus primarily inhibits mTORC1, whereas everolimus is more effective at inhibiting mTORC2 at clinically relevant concentrations [27]. Life span-enhancing effects of mTOR inhibitors seen in preclinical models are predominantly linked to mTORC1 inhibition, while inhibiting mTORC2 might be detrimental, as mTORC2 controls insulin-mediated suppression of hepatic gluconeogenesis [28]. Therefore, sirolimus has been selected for this study as it preferentially inhibits mTORC1 without significantly affecting mTORC2.

#### Rationale

Sirolimus (rapamycin) has the potential to restore mTORC1 balance by inhibiting its overactivation, which is commonly seen in aged muscles [29]. While mTORC1 is typically activated by branch-chain amino acids like leucine or by anabolic stimuli such as exercise, its chronic activation in older muscles leads to muscle atrophy rather than protein synthesis [29]. This atrophy is primarily due to the suppression of autophagy, highlighting the importance of mTORC1-regulated autophagy in aging muscle [29].

Weekly dosing with sirolimus (rapamycin) may provide alternating periods of mTORC1 activation (promoting protein synthesis) and inhibition (inducing autophagy), thereby improving muscle health. Given sirolimus (rapamycin)’s long half-life of 64 h in humans, weekly administration should be sufficient to create these beneficial cycles.

Combining exercise with weekly sirolimus dosing may offer synergistic benefits, enhancing muscle performance more effectively than exercise alone. This study aims to utilize a low-dose sirolimus protocol to balance therapeutic mTORC1 inhibition with minimal side effects, potentially offering a safe and effective strategy to boost muscle strength and endurance in the aging population.

## Objectives {7}

### Primary objective

The primary objective of this study is to evaluate the effect of once-weekly sirolimus (rapamycin) 6 mg on muscle strength and endurance in an older population engaged in a 13-week exercise program, compared to the exercise regimen alone. This evaluation will be primarily conducted through the 30-Second Chair-Stand Test (30CST), a measure specifically chosen for its relevance and sensitivity in assessing functional muscle performance in the elderly. The study seeks to establish that the addition of sirolimus (rapamycin), an inhibitor of the mechanistic target of rapamycin (mTORC1) pathway, does not adversely affect the exercise-induced improvements in muscle performance. Additionally, this study seeks to provide initial insights into whether intermittent modulation of mTORC1 signaling through this combined approach could enhance muscle performance beyond what is observed with exercise alone in older adults.

### Secondary objectives

There are several secondary objectives of this study as outlined below:

1. To investigate the safety and tolerability of low-dose rapamycin in exercising older adults.

Safety and tolerability measures utilized to investigate the incidence, relatedness, seriousness, and severity of adverse events include:

- Clinical laboratory tests (full blood count, U&Es, LFTs, HbA1c, lipids, serum IGF-1)
- Vital sign measurements
- Adverse events, serious adverse events, and events that impact exercise ability or dosing.

2. To evaluate change of muscle strength and muscle endurance from baseline to EOS Muscle strength and endurance measures will be evaluated by evaluating change from baseline in the:
  30-Second Chair-Stand Test (30CST)
  6-min walk test
  Handgrip strength

We will assess changes in upper body strength and cardiovascular endurance using grip strength and the 6-min walking test, respectively. The grip strength test will provide valuable insights into the impact of the intervention on muscular strength and function, critical for daily activities and autonomy in the elderly. The 6-min walking test will evaluate alterations in aerobic capacity and endurance, indicative of the participants’ ability to perform sustained physical activities.

3. Participant-reported outcomes
  Change from baseline in the self-reported measures of health status and quality of life as determined by the 36-Item Short Form Survey (SF-36) through EOS.

The validated SF-36 tool provides measurement of the intervention’s impact from the participants’ perspective, encompassing aspects such as pain, general health perceptions, physical functioning, emotional well-being, and social functioning.

- Participant self-reported physical activity, from baseline to EOS via participant diary.

#### Exploratory objectives

Additional exploratory objectives of this study are to investigate the potential effects of the combined exercise regimen and rapamycin treatment on biological aging markers and systemic inflammation, which are key factors in the aging process.

1. Epigenetic age assessment

Utilizing the advanced methodology of epigenetic clocks based on DNA methylation status, this study aims to explore the intervention’s impact on biological age.

2. Inflammation and aging (inflamm-aging)

Chronic inflammation is a recognized hallmark of aging, and managing the balance between pro-inflammatory and anti-inflammatory signals is crucial for healthy aging. By examining changes in hs-CRP, we aim to explore how the intervention influences systemic inflammation, thereby contributing to our understanding of the mechanisms underlying aging and longevity.

## Trial design {8}

This study is a randomized, double-blind, placebo-controlled trial designed to evaluate the effects of once-weekly sirolimus (rapamycin) 6 mg on muscle strength and endurance in older adults participating in a thrice-weekly exercise program for 13 weeks. Randomization will be carried out using block randomization with stratification for age with a 1:1 allocation to receive either once-weekly sirolimus (rapamycin) or a placebo in addition to their exercise regimen. The primary, exploratory objective is to evaluate whether this combined intervention is at least equivalent to regular exercise alone in enhancing muscle performance in older adults.

## Methods: participants, interventions and outcomes

### Study setting {9}

The trial will be conducted at the New Zealand Aotearoa Clinical Trials Trust, a single-center in New Zealand. The site is equipped with the necessary facilities for conducting clinical trials and will ensure participant safety and data integrity.

### Eligibility criteria {10}

#### Inclusion criteria

- Male or female aged ≥65 years and ≤85 years at the time of signing informed consent.
- BMI between ≤ 18 and ≥ 40 and a maximum weight of 120 kg at screening.
- Currently sedentary lifestyle or performing moderate exercises for less than 15 min and three times a week.
- Capable of providing written informed consent.
- Willing to swallow a #000 sized capsule.
- Willing and able to adhere to and comply with all study requirements and attend all study visits.
- Able to complete the 30-Second Chair Stand Test utilizing correct technique.
- Willing and able to accommodate and use an exercycle at home for the duration of study participation.

#### Exclusion criteria

- Anemia—Hg < 9.0 g/dl, leukopenia—white blood cells (WBC) < 3500/mm3, neutropenia—absolute neutrophil count < 2000/mm3, or platelet count—platelet count < 125,000/mm3.
- Planned surgery during the study period that impacts the ability to perform required study exercises.
- Any medical or psychological condition which, in the opinion of the investigator, may interfere with the participant’s ability to comply with the study and/or put the participant at significant risk.
- Impaired wound healing or history of a chronic open wound.
- Active infection at the time of signing consent or taking antibiotics or antifungal medications.
- Malignancy (except non-melanoma skin cancers, cervical carcinoma in situ) within the last 5 years.
- Known hypersensitivity, allergy, or any contraindication to sirolimus (rapamycin) or placebo (cellulose powder) or its excipients.
- Fibromyalgia, chronic fatigue syndrome/myalgic encephalomyelitis, breast implant illness, or other conditions that impact the participant's ability to perform the exercise program.
- Known congestive heart failure with New York Heart Association (NYHA) classification III or IV.
- Prescribed substances that inhibit or induce CYP3A4 and/or P-glycoprotein (P-gp).
- Current prescribed or reported cannabinoid use.
- COPD Global Initiative for Chronic Obstructive Lung Disease (GOLD) classification III or IV.
- Impaired renal function defined as glomerular filtration rate (eGFR) < 30.
- Type 1 diabetes or uncontrolled type 2 diabetes (defined as HbA1c ≥60 mmol/mol).
- Metformin, sirolimus (rapamycin), or rapalogs use within 6 months prior to baseline.
- Impaired hepatic function measured by alkaline phosphatase (ALP), alanine aminotransferase (ALT), albumin, or T. Bili whereby the levels are 1.5× greater than the normal upper limit.
- Any form of clinically relevant primary or secondary immune dysfunction or deficiency (e.g., X-linked agammaglobulinemia (XLA), common variable immunodeficiency (CVID)).
- Chronic oral corticosteroid or immunosuppressive medication use (e.g., Enbrel, Humira, methotrexate, ciclosporin).
- Participation in any other study (for 30 days) prior to or during this study.

### Who will take informed consent? {26a}

Prior to any study procedures being conducted, including screening assessments, the participant will be asked to provide informed consent to participate in this study. This will be done by Aotearoa Clinical Trials Trust. The following process will be followed for all participants:

- Potential participants will be provided with sufficient information and given time to consider participation in the study prior to providing informed consent.
- The investigator or investigator’s representative will explain the nature of the study fully to the potential participant, including risks and benefits, and answer any questions that they may have.
- Potential participants will be informed that their participation is voluntary, they may withdraw their consent at any time, and that they will be required to sign the participant information sheet and informed consent form (PISCF) that meets the requirements of ICH GCP, privacy, and data protection and has been approved by a New Zealand Health and Disability Ethics Committee (HDEC) committee.
- The participant’s medical record must include a statement that written informed consent was obtained prior to the participant being enrolled into the study and the date that written informed consent was obtained. The investigator or investigator’s representative will also sign and date the PISCF on the date consent was given by the participant.
- Participants will be re-consented to updated HDEC-approved versions of the PISCF during their participation in the study if applicable.
- A copy of the PISCF will be provided to the participant and the original stored in the investigators’ site file (ISF)
- Participants that are re-screened for any reason will be required to sign the PISCF again prior to their participation.

### Additional consent provisions for collection and use of participant data and biological specimens {26b}

Participants will be assigned a unique identifier by the sponsor. Any participant records or datasets that are transferred to the sponsor will contain the identifier only; participant names or any information which would make the participant identifiable will not be transferred.

The participant must be informed that their personal study-related data will be used by the sponsor in accordance with local data protection law. The level of disclosure must also be explained to the participant who will be required to give consent for their data to be used as described in the PISCF.

The participants must be informed that their medical records may be examined by quality assurance auditors and other authorized personnel appointed by the sponsor and by inspectors from regulatory authorities.

## Interventions

Explanation for the choice of comparators {6b}

### Choice of comparator

In this trial, the comparator chosen is a placebo, which is justified by the current understanding of sirolimus (rapamycin) and its role in mTORC1 inhibition. Sirolimus (rapamycin) is a well-studied compound with known effects on the mechanistic target of rapamycin (mTOR) pathway, which plays a significant role in cell growth, proliferation, and survival. By choosing a placebo, we aim to establish a clear and unbiased comparison to determine the efficacy and safety of intermittent sirolimus (rapamycin) dosing combined with an exercise regimen in older adults.

#### Rationale for placebo comparator

- Establishing baseline efficacy: using a placebo allows us to measure the true impact of sirolimus (rapamycin) on muscle strength and endurance by providing a baseline against which to compare the active treatment. This is particularly important as the primary objective is to evaluate the effect of once-weekly sirolimus (rapamycin) 6 mg on muscle strength and endurance in an older population engaged in a 13-week exercise program, compared to the exercise regimen alone.
- Safety profile**:** placebo use ensures that any adverse events can be accurately attributed to sirolimus (rapamycin), rather than confounding variables. This is crucial for understanding the safety and tolerability of rapamycin in an older population.
- Blinding and bias reduction**:** a placebo-controlled design helps maintain blinding, reducing bias in outcome assessment and participant reporting. This is essential for the internal validity of the study.

#### Comparator administration

Participants in the control group will receive matching placebo capsules containing cellulose, administered in the same manner as the sirolimus (rapamycin) capsules. This includes identical appearance, taste, and packaging to ensure blinding is maintained.

#### Clinical relevance

The clinical relevance of using a placebo comparator is underscored by the need to establish a clear cause-and-effect relationship between sirolimus (rapamycin) administration and improvements in muscle strength and endurance. Given the potential impact on clinical guidelines and therapeutic approaches for sarcopenia and aging, a rigorous comparison with a placebo is necessary.

This approach aligns with the ethical considerations of clinical trials, ensuring that participants receive a standard of care that is both safe and scientifically justified. The use of a placebo is appropriate given the current absence of established treatments targeting the mTORC1 pathway for muscle performance enhancement in older adults.

## Intervention description {11a}

### Exercise program

Both study groups will perform a standardized at-home exercise program, conducted three times per week. This program includes a combination of chair-stand exercises (part 1) and exercycle workouts (part 2), specifically tailored to suit the needs of older adults. To facilitate a uniform exercise experience and maintain consistency in the regimen, exercycles will be provided and set up in the participants’ homes.

Each exercise training day will commence with the chair-stand component (part 1), where participants are required to sit in a chair and then stand up as many times as possible within a 30-s timeframe. The 30CST forms part of the participants’ exercise program as well as provides practical application of the 30-Second Chair-Stand Test. The 30CST aims to enhance lower body strength and endurance, which are vital for daily activities and overall mobility.

Following the chair-stand exercise, participants will transition to using the exercycle (part 2). The exercycle workout is designed to improve cardiovascular fitness, leg strength, and overall endurance. This part of the exercise program is crucial for complementing the chair-stand activity, providing a balanced approach to muscle conditioning and aerobic exercise.

The following schedule of training will be completed by the participants each week from weeks 1 to 13. Day 1 of Week 1 must commence within 6 days of the Day 0 baseline visit.

**Table:** 

Weekly schedule	Exercise schedule
Day 1	Training program
Day 2	Rest day
Day 3	Training program
Day 4	Rest
Day 5	Training program
Day 6	Rest day
Day 7	Rest day

The training program for the day consists of two parts:

- Part 1: At the beginning of each training day, and before using the exercycle, participants must sit down in a chair and stand back up as many times as can be managed in a 30-s timeframe.
- Part 2: The use of the exercycle as follows:

**Table:** 

Week	Warm-up	Training	Cooldown
1	2 min @ 50RPM	10 min @70–80RPM, resistance level 1	2 min @ 45RPM
2	2 min @ 50RPM	15 min @70–80RPM, resistance level 1	2 min @ 45RPM
3	2 min @ 50RPM	20 min @70–80RPM, resistance level 2	2 min @ 45RPM
4	2 min @ 50RPM	25 min @70–80RPM, resistance level 2	2 min @ 45RPM
5	2 min @ 50RPM	25 min @70–80RPM, resistance level 3	2 min @ 45RPM
6	2 min @ 50RPM	25 min @70–80RPM, resistance level 3	2 min @ 45RPM
7	2 min @ 50RPM	25 min @70–80RPM, resistance level 4	2 min @ 45RPM
8	2 min @ 50RPM	25 min @70–80RPM, resistance level 4	2 min @ 45RPM
9	2 min @ 50RPM	25 min @70–80RPM, resistance level 5	2 min @ 45RPM
10	2 min @ 50RPM	25 min @70–80RPM, resistance level 5	2 min @ 45RPM
11	2 min @ 50RPM	25 min @70–80RPM, resistance level 5	2 min @ 45RPM
12	2 min @ 50RPM	25 min @70–80RPM, resistance level 5	2 min @ 45RPM
13	2 min @ 50RPM	25 min @70–80RPM, resistance level 5	2 min @ 45RPM

All warm-ups and cooldown phases should be completed with a resistance level 1 setting.

If participants are unable to complete the exercycle training program due to difficulty, the program can be adjusted whereby the resistance setting is lowered.

If a participant still cannot complete the training program, they should aim to ride for as long as they are able before moving to the cooldown phase.

Participants will record their exercise activities in the participant diary, and specifically for the exercycle sessions, the participants will include the length of training, the RPM, and the resistance setting.

### Sirolimus (rapamycin) administration

Participants assigned to the intervention group will receive sirolimus (rapamycin) at a dose of 6 mg per week, administered orally in the form of three capsules containing an unaltered 2 mg tablet of sirolimus (rapamycin) each. To measure treatment and dose adherence, the rapamycin will be blister-packed. Both used and unused blister packs must be returned to the site and collected at the 6-week and 13-week intervals for investigational product reconciliation.

### Placebo administration

Participants in the control group will receive matching placebo capsules, containing cellulose to fill the full capsule, administered in the same manner as the sirolimus (rapamycin).

## Criteria for discontinuing or modifying allocated interventions {11b}

Participants have the right to withdraw from the study at any time without providing a reason. However, the site should try to ascertain a reason when possible. The principal investigator or a study site team member can be notified by email, phone call, or in person. Participants may be withdrawn from the study at the investigator’s discretion if it is in the participants' best interests or due to significant participant non-compliance.

Discontinuation of treatment based on adverse events are to be discussed with the principal investigator. Discovery of treatment allocation will unblind the participant and the principal investigator and will lead to early withdrawal of the participant from the study.

## Strategies to improve adherence to interventions {11c}

One of the significant advantages of this study is the incorporation of a real-world, easy-to-follow exercise program using at-home exercycles. This approach makes the exercise regimen more accessible and sustainable for older adults, as it allows participants to perform the exercises in the comfort of their own homes without the need for frequent visits to a gym or clinical facility. This convenience is particularly beneficial for older adults who may face mobility challenges or have limited access to exercise facilities, thereby enhancing the likelihood of adherence to the exercise protocol.

To measure treatment and dose adherence, the rapamycin will be blister-packed. Both used and unused blister packs must be returned to the site and collected at the 6-week and 13-week intervals for investigational product reconciliation.

All participants will be contacted by site staff weekly to check that study interventions are being completed. Contact should be made by phone call. If contact via phone call has been attempted twice in a single week without success, a text and/or email may be utilized that week. Each attempt is to be documented in the participant’s source documents.

Weekly phone calls are to include a brief discussion about the participant’s compliance with treatment dosing and the exercise program. Adverse events are to be discussed and documented by the study site staff.

If a participant has specifically requested contact be via text or email, the request is to be documented, and the participant’s preferred method of contact may be utilized, provided that the participant is responsive to the alternative method of contact regarding study compliance and adverse events.

## Relevant concomitant care permitted or prohibited during the trial {11d}

If a participant fits the eligibility criteria, but is prescribed other medications, the participant should continue to take the prescribed medications at the prescribed dosages. The research team will inform the participant’s general practitioner (GP) of their participation in the study. Any changes to concomitant medications or any planned surgery should be discussed with the study site investigator immediately.

Medications known to interact with rapamycin are prohibited for the duration of the study.

This includes but is not limited to:

- Calcium channel blockers: diltiazem and verapamil
- Antifungal agents: clotrimazole, fluconazole, itraconazole, ketoconazole, and voriconazole
- Antibiotics: clarithromycin and erythromycin
- Gastrointestinal prokinetic agents: cisapride and metoclopramide
- Other drugs: bromocriptine, cimetidine, ciclosporin, danazol, letermovir, and protease inhibitors (e.g., for HIV and hepatitis C that include drugs such as ritonavir, indinavir, boceprevir, and telaprevir)
- Grapefruit juice
- Anticonvulsants: carbamazepine, phenobarbitone, and phenytoin
- Antibiotics: rifabutin and rifampicin
- Herbal preparations: St. John’s Wort (*Hypericum perforatum*, hypericin)
- Cannabinoids

## Provisions for post-trial care {30}

Participants who experience any adverse events related to the study will receive appropriate medical care. The study team will monitor participants throughout the trial to ensure their safety and well-being. Participants injured as a result of study procedures may be eligible for publicly funded compensation through the Accident Compensation Corporation (ACC) of New Zealand.

## Outcomes {12}

### Primary outcome

Our primary outcome is to evaluate the difference in the improvement of muscle performance, as measured by the 30-Second Chair-Stand Test (30CST), following a 13-week exercise regimen combined with weekly dosing of sirolimus (rapamycin) compared to exercise alone.

This assessment is crucial in establishing that the weekly administration of rapamycin, which inhibits the mechanistic target of the rapamycin (mTORC1) pathway, does not diminish the beneficial effects of exercise on muscle performance. While there is a potential for the sirolimus (rapamycin) group to exhibit a trend towards superiority in terms of exercise improvement, it is important to note that this study is not powered to conclusively determine superiority. Instead, our goal is to affirm that intermittent dosing of rapamycin alongside a regular exercise program will at least maintain, if not enhance, the muscle performance improvements that are typically observed with exercise alone in this demographic. This approach will provide valuable insights into the feasibility of incorporating rapamycin into exercise regimens for older adults, setting the stage for future studies that may be designed to explore the superiority of this combined intervention.

The rationale behind choosing the 30CST as our primary outcome measure lies in its relevance and sensitivity in assessing the functional muscle performance in older adults. By initiating an exercise regimen in participants who have not been regularly exercising—specifically, those not engaging in exercise that elevates heart rate for more than 15 min, three times a week—we anticipate observable improvements in muscle performance in the placebo group. This baseline improvement provides a critical comparative framework to evaluate the effects of rapamycin on exercise-induced muscle performance enhancements.

When measuring muscle performance, it is important to first define muscle strength, muscle power, and muscle endurance. Muscle strength refers to the amount of force a muscle can produce with a single maximal effort. Muscle power concerns work rate (work done per unit time) and is defined by the ability to exert a maximal force in as short a time as possible, as in accelerating, jumping, and throwing implements. Muscle endurance is the ability of muscles to exert force against resistance over a sustained period of time [30].

Compared to muscle strength, power concerns work rate (work done per unit time). In healthy older people, muscle power declines earlier and faster compared to muscle mass and strength. Leg power has been shown to be highly correlated with physical performance tests such as gait speed, chair-stand test, and stair-climb time, and several comparative studies have found that muscle power is a better predictor of mortality compared to muscle strength. Muscle power can be assessed across a range of muscle groups, but most often, the leg press and knee extension exercises are used to measure muscle power. The 30CST developed by Rikli and Jones is one of the most important physical performance clinical tests because it measures lower body power, balance, and endurance and relates it to the most demanding daily life activities. The 30CST has been widely used in many studies not only to evaluate functional fitness levels but also to monitor training and rehabilitation [30].

The original 30CST paper published in 1999 by Rikli and Jones provided the mean scores by age group [31]:

**Table:** 

Age group	Mean	Standard deviation
60–69	14.0	2.4
70–79	12.9	3.0
80–89	11.9	3.6

We also have data on the change in the 30CST after exercise. A study of 20 healthy women aged between 65 and 79 demonstrated that after 12 weeks of a combined exercise intervention program with extra emphasis on balance and muscle strength found that the 30CST increased by 13.5% (14.8 ± 4 to 16.8 ± 3.4) [32]. A subsequent trial of 29 older adults included both males and females with an average age of 76 demonstrated a 20% improvement in the 30CST after a 12-week training program [33].

### Secondary outcomes

#### Grip strength

Grip strength is a widely recognized and reliable measure of overall muscle strength and function. In geriatric populations, grip strength is particularly indicative of general health and has been correlated with important health outcomes, including mobility, nutritional status, and even mortality risk. By measuring grip strength, we can assess the specific impact of our intervention on upper body strength. This is crucial as upper body strength plays a significant role in daily activities and maintaining independence in older adults. Monitoring changes in grip strength throughout the study will provide valuable data on how the rapamycin and exercise regimen influence muscle strength and functional health.

#### 6-min walking test

The 6-min walking test is a practical and efficient measure of aerobic capacity and endurance. This test is particularly suitable for older adults as it does not require maximal exertion and reflects the physical capacity required for daily activities. By incorporating this test, we can evaluate the impact of the intervention on the participants’ cardiovascular fitness, lower body strength, and overall endurance. Improvements in the distance covered during the 6-min walking test can indicate enhanced functional endurance, a critical factor for the quality of life in older adults. This test complements the 30-Second Chair-Stand Test by providing a broader view of the participants’ functional mobility and endurance capabilities.

#### Epigenetic clocks

Epigenetic clocks are based on the methylation status of a set of genomic positions and provide an accurate age estimate in humans. Samples can be obtained from various cell types, including white blood cells, cheek cells obtained via a cheek swab, brain, the colon, and other organs (hence, it is considered a biomarker for the age of almost every part of the body). This sets the method apart from tests that rely on biomarkers of age that work in only one or two tissues, including the gold-standard dating procedure, aspartic acid racemization, which analyses proteins that are locked away for a lifetime in tooth or bone. In human DNA, methyl groups most often attach at CpG sites, where a cytosine precedes a guanine in the DNA. This process is catalyzed by at least three DNA methyltransferase (DNMTs). A typical human genome contains more than 28 million such sites. For this study, a genome-wide assessment of DNA methylation will be performed by TruDiagnostics via blood and saliva samples.

#### Chronic inflammation

Chronic inflammation through (over)stimulation of the immune system, also called inflamm-aging appears to be one of the hallmarks of aging while achieving healthy longevity lies in balancing the pro-inflammatory and anti-inflammatory signals. In order to measure this background inflammation, we will use hs-CRP.

#### 36-item short form survey (SF-36)

The 36-Item Short Form Survey (SF-36) is a commonly used, well-researched, self-reported measure of health. The SF-36 is often used as a measure of a person or population’s quality of life (QOL).

## Participant timeline {13}

### Schedule of assessments

**Table:** 

Procedures and assessments	Visit 1	Visit 2	Visit 2a	Visit 3	Visit 4
	Screening	Baseline	Phone call	Interim assessment	EOS
	Day -28 to -1	Day 0	Within 6 days of baseline	Day 42±7	Day 91 +7
Informed consent	x				
Review of subject eligibility	x	x			
Demographics^1^	x				
Medical history	x				
Auscultate the heart to check for murmurs	x				
Vital signs^2^	x	x		x	x
ECG	x				
Hematology and biochemistry	x^3^			x^4^	x^3^
DNA methylation (TruDiagnostics)		x			x
SF-36 questionnaire		x			x
Assessment of ability to complete 30-Second Chair-Stand Test	x				
Randomization		x			
Allocation of weekly medication^5^		x		x	
Confirm participant day 1			x		
Weekly participant contact			x^6^		
Adverse event collection		x	x	x	x
Distribute subject diary card		x		x	
Collect subject diary cards				x	x
30-Second Chair-Stand Test		x		x	x
Hand grip strength		x		x	x
6-min walk test		x			x

^1^Race, gender at birth, contact details, height, and weight

^2^Resting heart rate, blood pressure, and oxygen saturation

^3^Full blood count, eGFR, urea and electrolytes, LFTs, HbA1c, lipids, IGF-1, hs-CRP

^4^Full blood count, LFTs, lipids

^5^Allocation of investigational product for 6-week period, followed by a 7-week period

^6^Participants are to be contacted by phone each week. Participants who cannot be reached following two phone contact attempts in a week can be sent a text message or email

### Participant activities schedule

Weeks 1–13 exercise and dosing schedule.

Day 1 Week 1 must begin within 6 days of Day 0 (baseline visit).

**Table:** 

**Weekly schedule**	**Exercise schedule**	**Dosing schedule**
Day 1	Training program	No treatment
Day 2	Rest day	No treatment
Day 3	Training program	No treatment
Day 4	Rest	No treatment
Day 5	Training program	No treatment
Day 6	Rest day	Placebo/rapamycin in the morning
Day 7	Rest day	No treatment

## Sample size {14}

The sample size was selected primarily to satisfy logistic constraints of this feasibility study. As this study is not confirmatory, we do not predicate the sample size primarily on power considerations: arm sizes of 20, assuming no withdrawals, will provide 80% and 90% power, respectively, to detect fairly large Cohen’s effect sizes of 0.8 and 0.9, respectively, at a nominal significance level of 0.1.

In regard to safety, the arm sizes and post-randomization follow-up time will enable to detect rate ratios of adverse events between the sirolimus (rapamycin) and placebo arms varying between 2.4 and 3.7, dependent upon the rate in the placebo arm and the dispersion of the data. Sample sizes of 12 per arm have been indicated as sufficient for pilot or feasibility studies [34]. However, our feasibility objective of variance estimation makes it preferable to aim for a total sample size of 40, as a 95% confidence interval for the variance will then have expected half-width of less than half the true variance, providing a reasonably precise estimate.

### Recruitment {15}

Participants will be recruited through advertisements approved by the Health and Disability Ethics Committee (HDEC), including newspaper flyers, social media, radio, and television.

## Assignment of interventions: allocation

### Sequence generation {16a}

Randomization will be carried out using block randomization with stratification for age. The algorithm producing the schedule will be coded using an R script by the trial statistician, who will keep the blocking scheme secret. The code will be seeded with a randomly generated number and run by the statistician and transmitted to a third party. The resulting schedule will consist of a password-protected Excel spreadsheet containing participant study identifiers and corresponding allocation arms.

### Concealment mechanism {16b}

The third party will transmit the password-protected Excel schedule to the pharmacy service (CompoundLabs), which will prepare the placebo and sirolimus (rapamycin) blister packs accordingly and identify them with study identifiers. These identifiers will be assigned to participants sequentially in order of recruitment. Concealment is by remote randomization and sending the signal directly to the pharmacy.

### Implementation {16c}

The trial statistician will generate the allocation sequence. Study coordinators at the clinical trial site will enroll participants. Once enrolled, participants will be assigned to interventions by the pharmacy service (CompoundLabs) based on the allocation sequence provided. The study coordinators and participants will remain blinded to the group assignments throughout the study.

## Assignment of interventions: blinding

### Who will be blinded {17a}

The study participants, care providers, outcome assessors, and data analysts will be blinded to the treatment groups. The trial statistician and CompoundLabs, who are not involved in the study activities at the site, will perform the randomization and code the blister packs of the drugs according to the allocation sequence.

Both sirolimus (rapamycin) and placebo capsules will be identical in appearance, taste, and packaging. CompoundLabs will allocate the active product capsules or placebo capsules into the blister packs with unique codes.

Adverse events will be reported and managed by blinded staff to ensure that the blinding is not compromised. Measures will be taken to maintain blinding throughout the study, including the use of a separate team for data analysis that is not involved in participant care or outcome assessment.

### Procedure for unblinding if needed {17b}

Unblinding is permissible under specific circumstances where knowledge of the participant’s allocated intervention is essential for their safety and well-being. This may include serious adverse events where the clinical management of the participant requires knowledge of the treatment assignment.

In such cases, the following procedure will be followed:

1. Initiation of unblinding request: the treating physician or investigator will determine the necessity of unblinding and initiate the request. The request must be justified and documented, explaining the medical need for unblinding.
2. Consultation with the principal investigator: the principal investigator will review the request. If unblinding is deemed necessary, the principal investigator will proceed with the unblinding process.
3. Unblinding process: the principal investigator will contact the designated third party (CompoundLabs) responsible for maintaining the randomization code. CompoundLabs will provide the treatment allocation information directly to the principal investigator.
4. Documentation: the unblinding event, including the reason for unblinding, the date, and the personnel involved, will be documented in the participant’s case report form and study records.
5. Participant withdrawal: once unblinded, the participant will be withdrawn from the study to maintain the integrity of the trial. The participant will continue to receive appropriate medical care as required.
6. Notification: the relevant ethics committee will be notified of the unblinding event and provided with the necessary documentation.

By following this procedure, we ensure that unblinding is conducted ethically and only when absolutely necessary to protect the participant’s health and safety.

## Data collection and management

### Plans for assessment and collection of outcomes {18a}

After informed consent has been obtained (as described previously), a medical history, demographics, and subject eligibility will be reviewed:

1. Medical history review

At screening, relevant medical history relating to inclusion/exclusion criteria will be reviewed by the study site staff to ensure that the participant meets all the inclusion criteria and none of the exclusion criteria. It is not necessary to collect participant medical history outside of eligibility criteria, except for any event that has occurred within 30 days prior to the participant’s screening visit or any ongoing medical history for which the participant is currently receiving a concomitant medication.

2. Demographics

Participant demographics to be collected during the screening visit are as follows:

- Race
- Gender at birth
- Relevant contact details
- Height
- Weight

3. Eligibility review

All participants will be reviewed for eligibility during the screening period and again at base line visit prior to randomization to ensure that they meet the eligibility to participate in this study.

Participants will also be assessed for the ability to perform the 30-Second Chair Stand Test utilizing correct technique at screening for study eligibility

A physical examination of the participant will then commence:

1. Auscultation of the heart

At screening, the investigator or investigator’s delegate will auscultate the participant’s heart to review for heart murmurs that have not previously been detected.

2. ECG

A single 12-lead ECG will be performed at screening to assess for arrhythmias that would exclude the participant from participating in the study. No further ECGs are required during the study period unless the investigator or investigator’s delegate deems it necessary clinically.

3. Vital signs

All participants will have sitting blood pressure, pulse, and pulse oximetry performed at each on-site visit. If multiple readings are taken, the lowest should be recorded as data for the visit.

### Provision of exercycle

Following participant randomization at the baseline visit, the participant’s contact details (name, mobile number, and/or email address) will be provided to Rutherford Fitness, the exercycle provider. Rutherford Fitness will arrange a suitable time with the participant for the delivery of the exercycle to the participant’s place of residence. The exercycle model provided will be BK838M exercycles (with a 120-kg maximum weight allowance), for the 13-week period of the study exercise intervention period, unless the participants withdraw early from the study. Exercycles will be delivered and installed and instruction provided to the participant by Rutherford Fitness. Exercycles will be collected at the end of the 13-week study exercise period at a time agreed with the participant. Should a participant wish to purchase or continue the bike hire at their own cost at the end of the 13-week study exercise period, the participant must inform the investigator or investigator’s representative by the end of week 10. The site will provide contact details to Rutherford Fitness to make arrangements directly with the participant.

### Weekly contact

All participants will be contacted by site staff weekly to check that study interventions are being completed. Contact should be made by phone call. If contact via phone call has been attempted twice in a single week without success, a text and/or email may be utilized that week. Each attempt is to be documented in the participant’s source documents.

Weekly phone calls are to include a brief discussion about the participant’s compliance with treatment dosing and the exercise program. Adverse events are to be discussed and documented by the study site staff.

If a participant has specifically requested contact be via text or email, the request is to be documented, and the participant’s preferred method of contact may be utilized, provided that the participant is responsive to the alternative method of contact regarding study compliance and adverse events.

## Primary outcome

30-Second Chair Stand Test (30CST): the 30CST is the primary objective measure for the study, assessing muscle strength and endurance. During screening, participants will be briefed on the test requirements and assessed for their ability to perform the 30CST to determine eligibility. The test will be conducted at baseline (visit 2), mid-intervention (visit 3), and post-intervention (visit 4). Participants will receive instructions on the correct technique prior to each assessment to ensure consistency. The test procedure will be standardized, and participants can refer to the guidance video available at: https://www.physio-pedia.com/30_Seconds_Sit_To_Stand_Test.

## Secondary outcomes

### Hand grip strength

This measure will be recorded for all participants at visits 2, 3, and 4 using a dynamometer to assess upper body strength. The participant’s dominant hand will be tested to ensure consistent and reliable results.

### 6-min walk test

The 6-minute walk test (6MWT) will be completed at baseline (visit 2) and end of study (visit 4) for all participants according to a standardized protocol:

- Setup

The test should be conducted on a straight 30-m track, with 1-m marker points delineating the track.

Equipment required is 6MWT recording form, pulse oximeter, stop-watch or timer, chairs at each end of the 30-m course, sphygmomanometer and stethoscope for measuring BP, trundle wheel to ensure the 6MWT is accurately marked out, clip board and recording sheet, and portable oxygen if required.

A chair(s) should be available should a participant need to urgently sit down.

- Conducting the test:

The 6MWT test must be administered according to the standard protocol provided to the site, ensuring that the test is completed in a similar manner each time for consistency and accurate measurements.

### Hematology and biochemistry

Non-fasting blood samples will be collected at screening (visit 1) and the end of the study (visit 4) for laboratory testing, including full blood count, eGFR, urea and electrolytes, liver function tests (LFTs), HbA1c, lipids, IGF-1, and hs-CRP. An interim safety sample for full blood count, LFTs, and lipids will be collected at visit 3. If samples are lost or damaged, participants will be asked to provide another sample within a week, except during the screening period where a 28-day period applies.

### DNA methylation

At baseline (visit 2) and end of study (visit 4), all participants will provide a sample of whole blood to conduct DNA methylation testing with TruDiagnostic™, based in the United States of America (USA). Samples may be taken separately by finger-prick lancet onto the provided blood spot card, or whole blood can be taken from a no-additive sample tube already taken from the participant by venipuncture at the time. Preparation of the blood spot card will be according to the instructions provided by TruDiagnostic™. Collected samples will be stored at the site, at room temperature, and sent in batches to the TruDiagnostic™, no less than every 8 weeks to ensure that samples are viable when arriving at the analysis laboratory in the USA. Samples will be destroyed following analysis. Individual results will not be provided to participants as samples will be deidentified. If blood samples are lost or damaged and cannot be shipped for analysis, participants will be asked to provide another sample if the sample was lost within a week of providing the initial sample.

### SF36 questionnaire

Participants will be asked to complete theSF36 QoL questionnaire at baseline (visit 2) and end of study (visit 4). The questionnaire should be completed when the participant arrives at the study site for their appointment prior to any other activity occurring. The participant will be provided with the questionnaire on a tablet by the study staff and should complete the questionnaire unaided to ensure accurate answers from the participant.

### Participant diary

At baseline (visit 2) and visit 3, participants will be provided with a printed diary to complete daily. Information to be recorded will be the date, study required exercise program completed or any deviation from the assigned exercise program, other exercise completed that day not required by the protocol, study medication administration, and recording of any adverse event that has prevented the participant from completing the assigned exercise program or the assigned rapamycin dosing schedule.

### Data quality assurance

To promote data quality, assessors will receive training on standardized procedures for each outcome measure. Duplicate measurements will be taken when necessary to ensure accuracy. Data collection forms will be available in the protocol appendix for reference.

## Plans to promote participant retention and complete follow-up {18b}

To ensure high participant retention and complete follow-up, several strategies will be implemented throughout the study:

1. Regular communication
  ◦ Weekly contact will be maintained with all participants through phone calls, texts, or emails to discuss compliance with the exercise and medication regimen, address any concerns, and document adverse events. Each contact attempt will be documented in the participant’s source documents.
2. Convenience and support
  ◦ Exercycles will be delivered and installed at participants’ homes by Rutherford Fitness, ensuring they have the necessary equipment to complete the exercise program.
  ◦ Participants will receive detailed instructions and support for using the exercycle and completing the exercise regimen.
3. Monitoring and feedback
  ◦ Participants will be monitored regularly for adherence to the study protocol. Weekly phone calls will include discussions about compliance, and any issues will be addressed promptly.
  ◦ Participants will be encouraged to complete their daily diaries to record exercise activities, medication administration, and any adverse events. Compliance will be reviewed during the interim visit, and retraining will be provided as necessary.
4. Adverse event management
  ◦ Adverse events will be closely monitored and managed by blinded staff to ensure that participants’ safety is prioritized without compromising the blinding of the study.
  ◦ Participants experiencing significant adverse events will be consulted with the principal investigator to determine the best course of action, including the possibility of unblinding if necessary for safety reasons.
5. Data collection for withdrawn participants
  ◦ Efforts will be made to collect outcome data for participants who discontinue or deviate from the intervention protocols. This includes attempting to conduct end-of-treatment (EOT) assessments for those who withdraw early from the study.
  ◦ Participants who withdraw will be asked to return all study materials, including exercycles and study medication, and complete final assessments if they agree.
6. Participant engagement
  ◦ Regular updates on the study’s progress and findings (where appropriate) will be shared with participants to keep them engaged and informed about the importance of their contribution.

By implementing these strategies, the study aims to maintain high retention rates and ensure complete and accurate follow-up data.

### Data management {19}

Participants will be assigned a unique identifier by the sponsor. Any participant records or datasets transferred to the sponsor will contain the identifier only; participant names or any information that could make the participant identifiable will not be transferred.

Participants must be informed that their personal study-related data will be used by the sponsor in accordance with local data protection laws. The level of disclosure must be explained to the participant, who will be required to give consent for their data to be used as described in the participant information sheet and informed consent form (PISCF).

Participants must be informed that their medical records may be examined by quality assurance auditors and other authorized personnel appointed by the sponsor and by inspectors from regulatory authorities.

Information technology systems used to collect, process, and store study-related data are secured by technical and organizational security measures designed to protect such data against accidental or unlawful loss, alteration, unauthorized disclosure, or access.

Study participants will be provided with the option of receiving a summary of the study results.

Data entry and quality assurance

- Participant data relating to the study will be recorded on electronic case report forms (eCRFs) unless transmitted to the sponsor or designee electronically (e.g., laboratory data). The investigator is responsible for verifying that data entries are accurate and correct by physically or electronically signing the CRF.
- Guidance on completion of CRFs will be provided in the Data Management Plan and eCRF Completion Guidelines.
- Double data entry will be employed to ensure data accuracy, and range checks for data values will be implemented to identify and correct any discrepancies.
- Data quality will be promoted through predefined quality tolerance limits (QTLs) in the data management plan to identify systematic issues that can impact participant safety and/or the reliability of study results. These predefined parameters will be monitored during the study, and important deviations from the QTLs and remedial actions taken will be summarized in the clinical study report.

Data security and storage

- The sponsor or designee is responsible for data management of this study, including quality checking the data.
- The sponsor assumes accountability for actions delegated to other individuals, such as contract research organizations.
- Records and documents, including signed PISCFs, pertaining to the conduct of this study must be retained by the investigator for 10 years after study completion unless local regulations or institutional policies require a longer retention period. No records may be destroyed during the retention period without written approval from the sponsor. No records may be transferred to another location or party without written notification to the sponsor.
- Source documents provide evidence for the existence of the participant and substantiate the integrity of the data collected. Source documents must be filed at the investigator’s site and may not be distributed to the sponsor if there is any risk of identifying any particular participant.
- Data reported on the eCRF that are transcribed from source documents must be consistent with the source documents or discrepancies must be explained. All current medical records for participants must be available for review as part of study documentation. The investigator will describe documents comprising source documents before the study commences by documenting these on the source document location list. This list must be maintained throughout the study, along with the source documentation, to support the data that have been entered into the eCRF.
- The sponsor or designee will perform monitoring to confirm that the data entered into the eCRF by authorized site personnel are accurate, complete, and verifiable from source documents; that the safety and rights of participants are being protected; and that the study is being conducted in accordance with the currently approved protocol and any other study agreements, ICH GCP, and Medsafe Guideline on the Regulation of Therapeutic Products in New Zealand Part 11.

### Confidentiality {27}

All participant data will be treated with the utmost confidentiality. Personal information about potential and enrolled participants will be collected, shared, and maintained to protect confidentiality before, during, and after the trial.

Data collection

- Participants will be assigned a unique identifier by the sponsor. Any participant records or datasets transferred to the sponsor will contain the identifier only; participant names or any information that could make the participant identifiable will not be transferred.
- Personal information, including contact details, medical history, and study-related data, will be collected and stored in secure databases. These databases will be protected by technical and organizational security measures designed to prevent accidental or unlawful loss, alteration, unauthorized disclosure, or access.

Data sharing

- Access to personal information will be restricted to authorized personnel only. This includes study investigators, clinical site staff, and sponsor representatives who require access to carry out their responsibilities.
- Participants must be informed that their personal study-related data will be used by the sponsor in accordance with local data protection laws. The level of disclosure must be explained to the participant, who will be required to give consent for their data to be used as described in the participant information sheet and informed consent form (PISCF).
- Participants must be informed that their medical records may be examined by quality assurance auditors and other authorized personnel appointed by the sponsor and by inspectors from regulatory authorities.

Data maintenance

- Information technology systems used to collect, process, and store study-related data will be secured by technical and organizational security measures designed to protect such data against accidental or unlawful loss, alteration, unauthorized disclosure, or access.
- All records and documents, including signed PISCFs, pertaining to the conduct of this study must be retained by the investigator for 10 years after study completion unless local regulations or institutional policies require a longer retention period. No records may be destroyed during the retention period without written approval from the sponsor. No records may be transferred to another location or party without written notification to the sponsor.
- Source documents must be filed at the investigator’s site and may not be distributed to the sponsor should there be any risk of identifying any particular participant. Data reported on the eCRF that are transcribed from source documents must be consistent with the source documents or the discrepancies must be explained.

Data protection compliance

- Measures will be taken to protect personal information in accordance with the Health Information Privacy Code 2020 (New Zealand) and relevant international data protection regulations, including the General Data Protection Regulation (GDPR) where applicable.
- The sponsor or designee will ensure that all data protection and confidentiality policies are adhered to throughout the study.

By implementing these measures, the study aims to ensure the confidentiality and protection of participant data before, during, and after the trial.

### Plans for collection, laboratory evaluation, and storage of biological specimens for genetic or molecular analysis in this trial/future use {33}

Collection of biological specimens

- Biological specimens, including blood samples for hematology, biochemistry, and DNA methylation analysis, will be collected at specified time points throughout the study.
  - ◦ Hematology and biochemistry: non-fasting blood samples will be collected at screening (visit 1) and at the end of the study (visit 4) for laboratory testing. This will include a full blood count, eGFR, urea and electrolytes, liver function tests (LFTs), HbA1c, lipids, IGF-1, and hs-CRP.
  - ◦ DNA methylation: whole blood samples will be collected at baseline (visit 2) and at the end of the study (visit 4) for DNA methylation analysis. These samples can be collected via finger-prick lancet onto a blood spot card or by venipuncture into a no-additive sample tube.

Laboratory evaluation

- Hematology and biochemistry: samples will be analyzed according to local laboratory requirements, including full blood count, eGFR, urea and electrolytes, liver function tests, HbA1c, lipids, IGF-1, and hs-CRP. Safety blood samples, including full blood count, LFTs, and lipids, will also be collected at the interim visit (visit 3). Samples will be destroyed following analysis.
- DNA methylation: DNA methylation analysis will be conducted by TruDiagnostic™, based in the United States of America. Individual results will not be provided to participants as samples will be deidentified.

Storage of biological specimens

- Short-term storage: Collected samples will be stored at the clinical site at room temperature. Blood spot cards and other specimens will be kept in a secure, temperature-controlled environment to maintain their integrity until they are shipped for analysis.
- Long-term Storage and Shipping: Samples for DNA methylation analysis will be sent in batches to TruDiagnostic™ no less than every 8 weeks to ensure viability upon arrival at the analysis laboratory. Samples will be de-identified before shipping to maintain participant confidentiality. After analysis, the samples will be destroyed, and individual results will not be provided to participants.

Future use of specimens

- After analysis in this trial, all biological specimens will be destroyed.

Data management and confidentiality

- All biological specimens will be labeled with a unique participant identifier to ensure anonymity. Personal identifying information will be stored separately and securely, accessible only to authorized personnel.
- Data derived from the analysis of biological specimens will be stored in secure databases protected by technical and organizational security measures to prevent unauthorized access or disclosure.

### Statistical methods

#### Statistical methods for primary and secondary outcomes {20a}

As some of the feasibility issues are related to the efficacy analyses, they are described first. The efficacy analysis plan is intended to match the analysis plan of the full trial closely.

Efficacy analysis

- Analysis sets
  - ◦ Intention-to-treat (ITT) analysis set: This set will be used for the primary analysis and will include all randomized participants in their originally assigned groups.
  - ◦ Per-protocol (PP) analysis set: this set will be used for sensitivity analyses and will include participants who complete at least 75% of the exercise sessions and take at least 75% of the protocol-stipulated doses of sirolimus (rapamycin) or placebo.
- General inferential analysis plan
  - ◦ Outcomes at 13 weeks will be analyzed using regression models, adjusting for baseline values of the outcomes. Generalized linear models (GLM) will be employed, selected during a blind review of the data without knowledge of group allocation.
  - ◦ Final analyses will utilize the selected GLMs, and parametric assumptions will be addressed using the wild bootstrap method to estimate standard errors [35, 36]. For larger trials, standard sandwich estimators of the variance would be used in lieu of the bootstrap.
- Handling missing data
  - ◦ Multiple imputations will be used to create 15 completed datasets. The nominal significance level for this phase 2a study will be set at 0.1; however, in line with current thinking regarding preliminary studies, the results will deemphasize testing and emphasize point and interval estimation, producing confidence intervals for active arm effect having between 75% and 95% confidence levels in increments of 5 percentage points [27].
- Sensitivity analyses
  - ◦ Sensitivity analyses will be performed on the per-protocol analysis set and the complete-data-only set, excluding cases with missing data.

Safety analysis

- Safety analysis set: this set includes all randomized participants. Adverse events will be allocated on an as-treated basis:
  - ◦ Events occurring before cessation of treatment or completion of the trial will be assigned to the randomized arm.
  - ◦ Events occurring after cessation will be assigned to the placebo arm.
- Analysis of adverse events:
  - ◦ Counts of adverse events will be regressed on the allocation arm as described above in interaction with the graded severity using negative binomial regression.
  - ◦ Dichotomized relatedness to the intervention (classified as possible, probable, or definite) will be regressed on the allocation arm using a quasi-binomial framework with logarithmic link (relative risk regression) or a relative risk working model (e.g., Poisson with bootstrap estimation of the variance) to test for non-inferiority of the rapamycin arm. Standard errors will be estimated using the bootstrap, resampling participants. The modalities of non-inferiority will be detailed in the final analysis plan.

Feasibility analyses

- Screening and randomization rates: overall and weekly screening and randomization rates will be reported with Poisson-based 95% confidence intervals.
- Cessation and withdrawal rates: rates of drug treatment cessation, exercise program cessation, and study withdrawal will be reported and compared across randomization arms using a log-rank test.
- Residual variance estimation: the residual variance of each outcome will be estimated to inform the sample size and analytical design of the phase 3 trial.

Adherence to medication

- Adherence reporting: adherence to the medication will be reported by arm at 6 and 13 weeks using mean percentage of supply used and compared between arms using binomial regression with bootstrap-based standard errors and *p*-values [30].
- Causal effect estimation: the causal effect of the medication on the primary outcome will be estimated using two-stage residual inclusion estimation, with the randomized allocation as the instrumental variable [31].

## Interim analyses {21b}

No interim analyses are planned for this study and is based on several considerations:

- Study design and duration: the study is a relatively short-term phase 2a trial with a 13-week intervention period. Given the limited duration, the benefits of conducting interim analyses are minimal compared to the additional complexity and potential risks of unblinding or bias.
- Safety monitoring: continuous safety monitoring will be in place throughout the trial. Adverse events will be closely monitored and reviewed by the study team and an independent safety monitor to ensure participant safety without the need for formal interim analyses.
- Efficacy evaluation: the primary and secondary outcomes will be assessed comprehensively at the end of the study. Conducting interim analyses will not provide sufficient additional information to justify potential disruptions or modifications to the trial.

Continuous monitoring of participant safety will be maintained, with any serious adverse events or safety concerns being reported to the sponsor, ethics committee, and regulatory authorities as required.

### Methods for additional analyses (e.g., subgroup analyses) {20b}

Subgroup analyses: subgroup analyses will be conducted to explore the potential differential effects of the intervention across various participant characteristics and to provide a more detailed understanding of the efficacy and safety of sirolimus (rapamycin) in conjunction with exercise. These additional analyses are not limited to the following:

- Age groups: participants will be stratified into age groups (65–74 and 75–85 years) to assess whether the intervention’s effectiveness varies with age.
- Gender: the impact of the intervention will be evaluated separately for male and female participants to identify any gender-specific effects.
- Baseline fitness levels: participants will be grouped based on their baseline fitness levels, as measured by the 30-Second Chair-Stand Test (30CST) and other baseline assessments, to determine if initial fitness influences the intervention’s outcomes.
- Adherence to protocol: Subgroup analyses will consider adherence levels, comparing outcomes between participants who adhered strictly to the exercise and medication regimen (≥75% adherence) and those with lower adherence.

Adjusted analyses: adjusted analyses will be performed to account for potential confounding factors and to provide a more accurate estimate of the intervention's effects.

- Baseline covariates: the primary and secondary outcome analyses will be adjusted for relevant baseline covariates, such as age, gender, baseline fitness levels, and any other significant factors identified during the blind review of the data.
- Generalized linear models (GLM): these models will be used to account for repeated measures and the correlation between baseline and follow-up assessments.

### Methods in analysis to handle protocol non-adherence and any statistical methods to handle missing data {20c}

Definition of analysis populations

- Intention-to-treat (ITT) analysis set: this set will be used for the primary analysis and includes all randomized participants analyzed according to their original assigned groups, regardless of protocol adherence.
- Per-protocol (PP) analysis set: this set will be used for sensitivity analyses and includes participants who complete at least 75% of the exercise sessions and take at least 75% of the protocol-stipulated doses of sirolimus (rapamycin) or placebo.

Handling protocol non-adherence

- The primary analysis will follow the ITT principle, which means all participants will be analyzed as randomized. This approach maintains the benefits of randomization by including all participants, regardless of adherence, and is appropriate for estimating the effect of treatment in a real-world setting.
- Sensitivity analyses using the PP set will help to understand the effect of the intervention among those who adhered to the protocol. This analysis will exclude participants who fail to meet the adherence criteria, providing insights into the efficacy of the intervention when administered as intended.

Statistical methods to handle missing data

- Multiple imputation: missing data will be handled using multiple imputations, which helps to provide unbiased estimates by incorporating the uncertainty associated with missing data.
- Complete-case analysis: a complete-case analysis will be conducted, including only participants with no missing data, to compare and validate the results obtained from the multiple imputation method.
- Sensitivity analyses: sensitivity analyses will be conducted to assess the impact of different assumptions about the missing data. This may include:
  - ◦ Exploring the pattern and impact of missing data on the primary and secondary outcomes.
  - ◦ Comparing results from different imputation models to ensure robustness.

Quality assurance for data collection

- All efforts will be made to minimize missing data. This includes training for study staff on accurate and complete data collection, regular monitoring, and data verification processes.
- Data queries will be generated to address missing or potentially erroneous data. Participants will be asked to provide another sample within a week if blood samples are lost or cannot be processed.
- Participants will be guided during weekly phone calls to complete their daily diaries accurately. Compliance will be reviewed at the interim visit (visit 3), and retraining will be provided as necessary.

### Plans to give access to the full protocol, participant level-data and statistical code {31c}

Access to full protocol

- The full trial protocol will be made available to the public to ensure transparency and reproducibility of the research. It will be published as a supplementary document alongside the primary publication of the study results in a peer-reviewed journal.
- Additionally, the full protocol is accessible through the trial registry, Australia New Zealand Clinical Trials Registry (ANZCTR), under the registration number ACTRN12624000790549.

Access to participant-level data

- Participant-level data is anonymized to protect the privacy and confidentiality of study participants. Anonymized data sets will be made available to researchers upon reasonable request and after approval by the study’s sponsor.
- Requests for access to the participant-level data will need to include a detailed proposal outlining the research objectives and methods for data use. The proposal will be reviewed to ensure that it complies with ethical guidelines and data protection regulations.
- Access will be granted under a data-sharing agreement that stipulates the terms of use, including data protection measures and the requirement to acknowledge the original study.

Access to statistical code

- The statistical code used for data analysis will be made available to interested researchers to promote transparency and reproducibility. This will include scripts for data cleaning, analysis, and generation of results.
- The code will be shared through a public repository, such as GitHub or a similar platform, and linked in the primary publication of the study results.

Data management and confidentiality

- Source documents provide evidence for the existence of the participant and substantiate the integrity of the data collected. Source documents must be filed at the investigator’s site and may not be distributed to the sponsor should there be any risk of identifying any particular participant.
- Data reported on the electronic case report forms (eCRFs) that are transcribed from source documents must be consistent with the source documents or the discrepancies must be explained. All current medical records for participants must be available for review as part of study documentation. The investigator will describe documents comprising source documents prior to the study commencing by documenting these on the source document location list. This list must be maintained throughout the study, along with the source documentation, to support the data that have been entered into the eCRF.
- The sponsor or designee will perform monitoring to confirm that the data entered into the eCRF by authorized site personnel are accurate, complete, and verifiable from source documents; that the safety and rights of participants are being protected; and that the study is being conducted in accordance with the currently approved protocol and any other study agreements, ICH GCP, and Medsafe Guideline on the Regulation of Therapeutic Products in New Zealand Part 11.

### Oversight and monitoring

#### Composition of the coordinating center and trial steering committee {5d}

Sponsor

- Dr. Brad Stanfield
  - ◦ Dr. Brad Stanfield designed and set up the trial. He is responsible for the overall conceptualization and design of the study.
  - ◦ As the sponsor, Dr. Brad Stanfield Ltd oversees the trial, ensuring that it is conducted according to the protocol and regulatory requirements.
  - ◦ Dr. Brad Stanfield will also act as the Data Monitoring Committee (DMC), Trial Steering Committee, and Endpoint Adjudication Committee, providing recommendations regarding the continuation, modification, or termination of the trial and ensuring participant safety and data integrity.

Principal investigator and research physician

- Dr. Joanna Wojciechowska, Aotearoa Clinical Trials Trust
  - ◦ Responsible for conducting the trial, overseeing the day-to-day management of the study, ensuring adherence to the protocol, and safeguarding participant safety.
  - ◦ Reports serious adverse events to the ethics committee and regulatory authorities.

Coordinating Center

- ◦ Provides organizational support for the trial, coordinating all trial activities, maintaining communication with all trial sites, and ensuring compliance with regulatory requirements.
- ◦ Meets weekly to discuss trial progress, address any issues, and plan upcoming activities.

Contract research organization

- Project management: BioValeo Ltd.
  - Project manages and oversees the conduct and delivery of the study in accordance with NZ Medsafe Guideline on the Regulation of Therapeutic Products in New Zealand Part 11 and International Conference on Harmonisation Good Clinical Practice (ICH GCP) guidelines.
  - Responsible for contributing to study quality by design, study planning, and documentation generation.
  - Project management will include the oversight and management of vendors, study risks, and issues.
  - Overall responsible for study team management, study start-up, conduct and follow-up activities until study archiving, ensuring compliance with regulatory requirements and the protocol.
- Clinical monitoring: BioValeo Ltd.
  - Monitors the study in accordance with NZ Medsafe Guideline on the Regulation of Therapeutic Products in New Zealand Part 11 and International Conference on Harmonisation Good Clinical Practice (ICH GCP) guidelines.
  - A monitor will visit the site according to the monitoring plan and data will be regularly reviewed remotely, with regular and consistent communication maintained with the study site.
  - Periodic monitoring visits will be made to the site during the study to ensure the investigator obligations are being followed, the site facilities remain acceptable, HDEC has been notified of approved protocol changes as required, complete study records are being maintained, appropriate and timely reports have been made to the sponsor and HDEC, study drug inventory is controlled, and the investigator is carrying out all agreed activities.
  - A risk-based monitoring approach will be implemented, combining on-site and centralized monitoring to ensure participant protection and the quality and integrity of clinical trial data while promoting efficiency. On-site monitoring will be performed periodically based on the findings of the last on-site visit and centralized monitoring findings. During on-site monitoring, a percentage of the data will be reviewed (source document review and source document verification), and each participant’s source documents and data discrepancies will be queried.
- Data management: BioValeo Ltd.
  - Responsible for maintaining the trial IT system, data entry, and data verification.
  - Ensure that participant data related to the study is recorded on electronic case report forms (eCRFs) unless transmitted electronically (e.g., laboratory data).
  - The investigator verifies that data entries are accurate and correct by physically or electronically signing the CRF.
  - Meets biweekly to review data management activities and address any data-related issues.

Ethics committee reporting

- Principal investigator
  - ◦ Reports serious adverse events (SAEs) that occur while the participant is actively participating in the research study to the Health and Disability Ethics Committee (HDEC) annually.
  - ◦ Ensures that follow-up reporting of SAEs continues until the event has resolved.

Regulatory authority reporting

- Principal investigator
  - ◦ Reports serious adverse events related to approved medicines, such as Rapamycin, via the Medsafe website https://www.medsafe.govt.nz/safety/report-a-problem.asp.
  - ◦ Institutes appropriate diagnostic and therapeutic measures and keeps the participant under observation for as long as medically indicated.

Trial steering committee and endpoint adjudication committee

- Dr. Brad Stanfield
  - ◦ As this is an investigator-led study by a clinician, Dr. Brad Stanfield will act as the trial steering committee and endpoint adjudication committee.
  - ◦ Roles and responsibilities:
    - ▪ Trial steering committee: provides overall supervision of the trial, ensuring that it is conducted according to the protocol and regulatory requirements. Reviews trial progress, addresses significant issues, and makes decisions regarding protocol amendments or trial termination if necessary. Meets quarterly to review trial status, discuss interim findings, and provide strategic guidance.
    - ▪ Endpoint adjudication committee: reviews and adjudicates clinical endpoints to ensure unbiased and accurate assessment of the primary and secondary outcomes. Meets as needed, based on the occurrence of endpoints requiring adjudication.
  - ◦ To maintain ethical standards, it is important to note that Dr. Brad Stanfield is completely separate from Aotearoa Clinical Trials Trust, which is responsible for conducting the trial. This separation ensures the integrity and ethical conduct of the trial.

### Composition of the data monitoring committee, its role and reporting structure {21a}

Composition and role

- As this is an investigator-led study by a clinician, Dr. Brad Stanfield will act as the Data Monitoring Committee (DMC).
- The role of the DMC is to monitor safety data and overall trial conduct, providing recommendations regarding the continuation, modification, or termination of the trial. The DMC ensures that participant safety is prioritized and that the trial is conducted in accordance with the approved protocol.

Independence and competing interests

- As Dr. Brad Stanfield is the sponsor and acting DMC, this setup is not independent from the sponsor. However, the dual role aims to maintain close oversight of the trial and rapid response to any safety concerns.
- The protocol acknowledges this potential conflict of interest and emphasizes the commitment to participant safety and data integrity.
- Dr. Brad Stanfield is completely separate from Aotearoa Clinical Trials Trust, which is responsible for conducting the trial. This separation is maintained to ensure the integrity and ethical conduct of the trial.

### Adverse event reporting and harms {22}

Plans for collecting adverse events

- All adverse events (AEs) will be collected for this trial, including AEs leading to cessation or interruption of the study medication dosing schedule or interruption of the exercise program.
- Adverse events will be solicited during scheduled study visits and through spontaneous reports from participants.

Assessment of adverse events

- An assessment will be made by the investigator or designee as to the severity and relatedness of the event.
- Severity will be classified as mild, moderate, or severe based on the impact on the participant's health and daily activities.
- Relatedness to the study intervention will be categorized as unrelated, possibly related, probably related, or definitely related.

Reporting of serious adverse events (SAEs)

- Any SAE, as defined by this protocol, starting at the time of randomization (day 0), is to be reported to the sponsor within 24 h of the site staff becoming aware of the event.
- Reporting will occur by completing the relevant page within the electronic case report form (eCRF) by the investigator or delegated individual.
- If the eCRF is not available at the time of the event, the investigator or delegated team member will contact the medical monitor directly.

Management of adverse events

- The responsible investigator should institute appropriate diagnostic and therapeutic measures and keep the participant under observation for as long as medically indicated.
- Follow-up reporting of SAEs should continue until the event has resolved or stabilized.

Documentation and communication

- All AEs and SAEs will be documented in the participant’s medical records and reported in accordance with regulatory requirements.
- The sponsor will ensure that all relevant regulatory authorities and ethics committees are notified of SAEs as required by local regulations and guidelines.

Handling of unintended effects

- Any unintended effects of the trial interventions or trial conduct will be reported and managed according to the same procedures as for AEs and SAEs.
- Regular safety reviews will be conducted by the data monitoring committee (DMC) to identify and address any emerging safety concerns promptly.

Protocol-specific details

- Periodic monitoring visits will be conducted to ensure compliance with adverse event reporting requirements.
- A risk-based monitoring approach will be employed, combining on-site and centralized monitoring to ensure participant protection and data integrity.

### Frequency and plans for auditing trial conduct {23}

Frequency of audits

- Audits will be conducted periodically throughout the trial to ensure compliance with the protocol, Good Clinical Practice (GCP) guidelines, and regulatory requirements. The frequency of audits will be determined by the sponsor’s monitoring plan, which will be outlined in the study-specific monitoring plan.

Procedures for auditing

- Audits will involve a detailed review of trial documentation, including source documents, electronic case report forms (eCRFs), informed consent forms, and any other relevant trial records.
- Auditors will verify that data entries in the eCRFs are accurate and consistent with source documents and ensure that any discrepancies are appropriately addressed.
- The audit process will include reviewing the informed consent process, the handling and reporting of adverse events, drug accountability, and compliance with the approved protocol.
- Audits will also assess the implementation of risk-based monitoring strategies, such as the combination of on-site and centralized monitoring described in the protocol.

Independence of the auditing process

- The auditing process will be independent of the investigators and the sponsor to maintain objectivity and impartiality.
- External auditors appointed by the sponsor will conduct the audits. These auditors will not be involved in the day-to-day conduct of the trial or its management, ensuring an unbiased evaluation of trial conduct.

Reporting structure

- Findings from the audits will be documented in detailed audit reports, which will be submitted to the sponsor, Dr. Brad Stanfield Ltd.
- Any significant issues identified during the audits will be communicated immediately to the sponsor and relevant regulatory authorities if necessary.
- The sponsor will be responsible for addressing audit findings and implementing corrective actions to resolve any issues.

Additional relevant information

- As part of the risk-based monitoring approach, the combination of on-site and centralized monitoring will promote efficiency and ensure participant protection as well as the quality and integrity of clinical trial data.

### Plans for communicating important protocol amendments to relevant parties (e.g., trial participants, ethical committees) {25}

Any amendments to the protocol will be submitted to the Health and Disability Ethics Committee (HDEC) for review and approval before implementation. This includes changes that may impact participant safety, alter the study’s design or procedures, or affect the integrity of the data collected.

Communication to relevant parties

- Investigators: all investigators involved in the study will be promptly informed of any protocol amendments. This communication will include detailed descriptions of the changes and the rationale behind them. Investigators will receive updated versions of the protocol and any other relevant documents.
- Ethics committees (HDEC): the HDEC will review and approve all protocol amendments before they are implemented. Detailed documentation of the amendments, including the reason for the changes and their potential impact on the study, will be provided.
- Trial participants: participants will be informed of any significant changes that may affect their willingness to continue in the study. This includes changes to eligibility criteria, outcomes, and study procedures. Participants will receive updated informed consent forms if necessary, and their continued participation will be re-consented as needed.
- Trial registries: updates to the Australia New Zealand Clinical Trials Registry (ANZCTR), will be made to reflect any protocol amendments. This ensures that the public record of the trial remains accurate and up-to-date.
- Journals and regulators: any significant protocol amendments that may impact the publication of results or regulatory oversight will be communicated to relevant journals and regulatory authorities. This ensures transparency and compliance with reporting standards.

Documentation and record-keeping

- All protocol amendments and related communications will be documented and maintained in the trial master file. This includes approval letters from the HDEC, updated protocol versions, and records of communications with investigators, participants, and other relevant parties.

Additional relevant information

- The sponsor, Dr. Brad Stanfield Ltd., will oversee the communication process to ensure that all relevant parties are informed in a timely and accurate manner.
- Regular meetings and updates with the coordinating center, Aotearoa Clinical Trials Trust, will ensure that any protocol amendments are seamlessly integrated into the trial’s conduct.

#### Dissemination plans {31a}

Publication and communication of trial results

- Proprietary information: all information resulting from this study is the Proprietary Information of Dr. Brad Stanfield Ltd. and Vitasang Ltd. Dr. Brad Stanfield Ltd. and Vitasang Ltd. will have final and sole control over the content of any publication.
- Publication control: Dr. Brad Stanfield, the clinician who set up the study, plans to publish and present the findings in their entirety. The coordinating investigator and sub-investigators may make presentations on the study or publish results of the study at their site, but only after the results of the study have been published, or with the prior approval of Dr. Brad Stanfield Ltd.

Dissemination to participants and healthcare professionals

- Participants: participants will be informed of the trial results through a summary of the findings provided in layman's terms. This summary will be distributed to participants after the study results have been published.
- Healthcare professionals: results will be communicated to healthcare professionals through presentations at scientific conferences, seminars, and other relevant medical meetings. Additionally, healthcare professionals will have access to published articles in peer-reviewed journals.

Public and wider audience

- Public disclosure: trial results will be made publicly available through publication in peer-reviewed journals. Abstracts and presentations at scientific conferences will also be used to disseminate findings to a broader audience.
- Trial registries: results will be reported in results databases such as the Australia New Zealand Clinical Trials Registry (ANZCTR), ensuring transparency and accessibility to the public and other researchers.

Data sharing arrangements

- Access to data: anonymized data sets may be made available to researchers upon reasonable request and after approval by the study’s Sponsor. Requests for access to the data will need to include a detailed proposal outlining the research objectives and methods for data use.
- Data sharing agreement: access will be granted under a data-sharing agreement that stipulates the terms of use, including data protection measures and the requirement to acknowledge the original study.

Publication restrictions

- Sponsor’s discretion: the sponsor may, at their discretion, require the removal of any proprietary information from any publication or presentation.

Additional relevant information

- Ethical standards: the dissemination of results will adhere to ethical standards and guidelines to ensure that the findings are communicated responsibly and accurately.
- Collaboration: collaboration with other researchers and stakeholders will be encouraged to promote the broader application and understanding of the study findings.

## Discussion

The primary rationale for this study is to explore the potential benefits of combining intermittent sirolimus (rapamycin) administration with resistance training in older adults, aiming to improve muscle strength and endurance. This intervention targets a critical need in aging populations, where declining muscle mass and strength significantly contribute to frailty and decreased quality of life. By alternating periods of mTOR inhibition through rapamycin and activation via exercise, this study will explore a novel approach to enhancing muscle strength and endurance in the aging population. By investigating this combined approach, the study seeks to provide valuable insights into interventions that could enhance muscle performance and overall health, potentially leading to broader applications in geriatric care and preventative health strategies.

One of the key strengths of this study is its design, which seeks to mimic a real-world, easy-to-follow exercise protocol. Participants will use at-home exercycles, making the exercise regimen more accessible and sustainable. This approach not only enhances the feasibility of the intervention but also increases the likelihood of adherence, as participants can integrate the exercise into their daily routines without the need for frequent visits to a gym or clinical facility. The use of at-home exercycles is particularly beneficial for older adults, who may face mobility challenges or have limited access to exercise facilities.

Despite these strengths, the study faces several practical and operational challenges. Recruiting and retaining older adults (aged 65–85) for a 13-week intervention trial can be difficult due to health issues, mobility limitations, and other personal commitments common in this age group. To address these challenges, the study will implement multiple recruitment strategies, and retention will be promoted through regular follow-up calls, personalized reminders, and support from study staff to encourage ongoing participation.

Ensuring adherence to the protocol, both in terms of the sirolimus (rapamycin) dosing schedule and the supervised resistance training program, is critical for the validity of the study outcomes. Participants will receive detailed instructions and training, regular monitoring and support, and compliance aids such as medication diaries and exercise logs. Weekly phone calls and regular visits will help monitor adherence and promptly address any issues that arise.

Safety monitoring is another crucial aspect of the trial, given the potential for drug interactions and age-related health conditions in the older population. The study has a robust safety monitoring plan in place, including regular health assessments, close monitoring of adverse events, and immediate reporting of serious adverse events. The principal investigator and medical monitor will ensure participant safety through continuous oversight and prompt intervention if needed.

Accurate and complete data collection is essential for the reliability of the study results. The logistical challenges include ensuring data consistency and handling missing data. The study employs electronic case report forms (eCRFs) to streamline data entry and minimize errors. Data management will include regular data verification, the use of multiple imputation methods for handling missing data, and thorough training for data entry personnel.

An interim analysis is not planned for this study due to its relatively short duration and focused scope. Continuous safety monitoring will suffice to ensure participant protection, and the final analyses will provide comprehensive insights into the study outcomes.

Effective coordination and communication among the study team, participants, and external parties such as ethics committees and regulatory bodies are essential for smooth trial conduct. Regular meetings and updates will be held to ensure all team members are informed and aligned. Clear communication channels will be maintained with the ethics committee (HDEC), regulatory authorities, and other relevant stakeholders.

The study has several limitations. Its short duration (13 weeks) may limit the ability to observe long-term effects of sirolimus (rapamycin) combined with exercise. Additionally, the relatively small sample size (*n* = 40) may affect the generalizability of the results. Larger, long-term studies will be needed to confirm findings and assess broader applicability.

Despite these limitations, the insights gained from this trial will inform the design of larger, more comprehensive studies to evaluate the long-term benefits and safety of sirolimus (rapamycin) in older adults. Future research may explore different dosing regimens, combinations with other interventions, and the effects on additional health outcomes such as cognitive function and cardiovascular health.

In conclusion, this study aims to provide valuable insights into the potential benefits and safety of intermittent sirolimus (rapamycin) administration combined with resistance training in older adults. By addressing practical and operational challenges, the study seeks to contribute to the understanding of interventions that may improve muscle performance and overall health in aging populations.

## Trial status

The current protocol version is V1.0, dated 17 June 2024. Recruitment for the trial is due to begin on 23 July 2024. It is anticipated that recruitment will be completed by 30 September 2024.

## Data Availability

The final trial dataset will be accessible to the principal investigators and authorized study personnel at Aotearoa Clinical Trials Trust. Anonymized datasets will be made available to researchers upon reasonable request, subject to approval by the study’s sponsor, Dr. Brad Stanfield Ltd. Requests for access to the data will need to include a detailed proposal outlining the research objectives and methods for data use. Access will be granted under a data-sharing agreement that stipulates the terms of use, including data protection measures and the requirement to acknowledge the original study.
